# Health Tract, No. 288

**Published:** 1867-05

**Authors:** 


### MALARIA AND MIASM.

Malaria is literally bad air, and includes all kind of air not entirely pure. Miasm is a something which arises from the earth under certain conditions, and is of such a subtle nature, that chemical analysis has hitherto failed to detect its presence; and yet, it is so malignant in its effects in certain localities, that a few hours exposure to its inhalation, is sufficient to induce malignant fevers and deadly diseases, fatal within a few days. This miasm abounds most fatally on the banks of sluggish streams and stagnant waters, such as on the bayous of the South and the prairies of the West, and all other undrained localities. But miasm can exist under one set of circumstances only, heat above eighty degrees, with moisture, must act on vegetable matter, until it begins to decay; when decay commences, then the poisonous virus begins to rise and induce its deadly effects. During 1866, some facts were published in Illinois, and simultaneously in France, indicating that the offending material in what was called a miasmatic atmosphere, was possessed of life; a French lady avering in a scientific paper that it was a vegetable product, capable of multiplying itself with wonderful activity; while a physician of Chicago was at the same time carrying on a series of experiments, and making observations, seeming to prove that it was of animal origin, a microscopic insect made clearly visible. It was stated that if a person breathed a miasmatic air, the living thing could be detected in the saliva, and that if a quantity of the air was conveyed to a distance, and was put in a room where it could be breathed, that miasmatic disease, fever, diarrhoea, dysentery, &c., were produced. But whether dead matter or a vegetable product or a living animal, the clearly ascertained laws of miasm remain the same, and one great practical fact stands out with striking prominence, that there should be a good drainage under and around every human habitation, and that no dwelling near stagnant water can be healthful.

